# Low E2F2 activity is associated with high genomic instability and PARPi resistance

**DOI:** 10.1038/s41598-020-74877-1

**Published:** 2020-10-21

**Authors:** Jonathan P. Rennhack, Eran R. Andrechek

**Affiliations:** 1 Department of Physiology, Michigan State University, East Lansing, MI USA; 2 Department of Physiology, Michigan State University, 2194 BPS Building, 567 Wilson Road, East Lansing, MI 48824 USA

**Keywords:** Breast cancer, Cancer genomics, Cancer models

## Abstract

The E2F family, classically known for a central role in cell cycle, has a number of emerging roles in cancer including angiogenesis, metabolic reprogramming, metastasis and DNA repair. E2F1 specifically has been shown to be a critical mediator of DNA repair; however, little is known about DNA repair and other E2F family members. Here we present an integrative bioinformatic and high throughput drug screening study to define the role of E2F2 in maintaining genomic integrity in breast cancer. We utilized in vitro E2F2 ChIP-chip and over expression data to identify transcriptional targets of E2F2. This data was integrated with gene expression from E2F2 knockout tumors in an MMTV-Neu background. Finally, this data was compared to human datasets to identify conserved roles of E2F2 in human breast cancer through the TCGA breast cancer, Cancer Cell Line Encyclopedia, and CancerRx datasets. Through these methods we predict that E2F2 transcriptionally regulates mediators of DNA repair. Our gene expression data supports this hypothesis and low E2F2 activity is associated with a highly unstable tumor. In human breast cancer E2F2, status was also correlated with a patient’s response to PARP inhibition therapy. Taken together this manuscript defines a novel role of E2F2 in cancer progression beyond cell cycle and could impact patient treatment.

## Introduction

Breast cancer remains the leading cause of cancer related deaths in women. This is largely due to two factors, metastasis and the heterogeneity of breast cancer. While metastasis to distal sites is responsible for mortality, the difficulty of treating a heterogeneous disease is one of the primary factors allowing that progression to occur. The heterogeneity of breast cancer is evident in several facets, including histological subtypes, progression and response to treatment. Underlying this diversity are the unique genomic alterations, methylation patterns and the resulting gene expression differences that are recognized in the PAM50 classification system^1,2^. Each of the subtypes present (Luminal A/B, Basal, Claudin Low, HER2 + ve and normal like) have a unique transcriptomic profile, resulting in the dysregulation in key proteins in breast cancer, including the alteration of the E2F family of transcription factors^3–7^.

The E2F family of transcription factors is composed of nine unique family members (E2F1, E2F2, E2F3a, E2F3b, E2F4, E2F5, E2F6, E2F7, and E2F8)^8–10^. Classically they have been divided into family members that activate transcription (E2F1, E2F2, and E2F3a) and repressors of transcription (E2F3b, E2F4, E2F5, E2F6, E2F7, and E2F8). These definitions have recently become less clear, with each family member functioning in both activating and repressing roles depending on the tissue and developmental context^11,12^.

The activators specifically exist in a bound state to Rb at regions containing an E2F motif in a quiescent cell and transcription of controlled genes is repressed. However, upon a growth signal such as myc, cyclin D levels rise which in turn lead to the activation of cyclin dependent kinases, such as CDK4 and 6. Rb is phosphorylated by CDK4 and 6 and is released from the E2F complex at the gene promoter. This allows transcriptional machinery to bind the region and allows for increased transcription of E2F controlled genes^11,13–15^.

The E2F family members are highly conserved with E2F2 containing 46% homology to E2F1^16^. As a result, family members bind a conserved motif with gene specificity contributed by cofactors^12,17,18^. With regards to the development of the lens in an Rb null embryo E2F1, E2F2, and E2F3 loss all have different phenotpyes indicating a unique role for each family member^19^. This has also been shown to be true in cancer, with the loss of each E2F family member having independent effects on metastasis in a variety of mouse models^3,4,6^.

While, this role is not governed by the DNA binding sequence, evidence shows that protein co-factors control the specificity of the E2F family members. Work has shown the binding of co-factors such as YY1 and RYBP to direct E2F2 to specific sites not bound by E2F1 including Cdc6^20^. Due to the binding of YY1, RYBP and other cofactors E2F2 has been shown to perform many independent functions from other E2F family members including key roles in the development of inflammation in rheumatoid arthritis through STAT1 and PI3K^21^. Interestingly, both of these pathways have important roles beyond cell cycle and indicate the involvement of the E2Fs and E2F2 specifically beyond cell division.

The role of the E2F family has widely been described in cell cycle where the members regulate the G1/S checkpoint in response to Cyclin D levels^22,23^. However, beyond the G1/S checkpoint de-regulation E2Fs have a number of emerging roles in cancer^24^. This includes roles in other aspects of cancer progression including angiogenesis^25^, metabolic reprograming^26^, and apoptosis^27,28^. Indeed, numerous accounts detail the role of the activators in metastasis of human breast cancer as well as mouse models of the disease^3,4,6,29–31^.

An additional emerging role for the E2Fs has been in the regulation of genomic stability. Specifically, the role of E2F1 has been well defined with both transcriptional and non-transcriptional roles in DNA repair^32^. In response to DNA damage, E2F1 undergoes post translational phosphorylation by ATM^33^, leading to protein stabilization and increased expression of repair proteins. In addition to the transcriptional role in DNA repair, E2F1 is physically recruited to sites of damage. During cases of double stranded breaks^34^ or UV damage^35^ it was observed that E2F1 formed foci with other damage induced proteins at the site of DNA damage. It has been shown the E2F1 is required for the efficient recruitment of other repair proteins including XPA/XPC^35^ and NBS1^36^. Due to the well-defined role of E2F1 in instability and repair, we wanted to understand the specific role of other activator E2Fs in this process. An role for E2F2 specifically has been identified but it is poorly described. It has also been shown that E2F2 is transcriptionally upregulated in response to DNA damage and has been shown to complex with Rad51 and sites of DNA damage in neuronal cells^37^. It is unclear how this is preserved in the context of cancer or the specific mechanism of repair.

Within the context of cancer, the amplification of the centrosome within a cell leads to defects in cellular segregation and DNA replication, which in turn leads to the single nucleotide variants, copy number alterations, and translocations characteristics. Importantly, activator E2Fs have been shown to be associated with centrosome amplification^38^. It is through this amplification of the centrosome that it is believed E2Fs contribute to the DNA instability associated with their misregulation. However, the mechanism and specific E2Fs involved in this process remains undefined. Specifically, it is unclear what role E2F1, E2F2, and E2F3a play in this process. Together, there is an emerging role for the activator E2Fs role in maintaining genomic integrity, but only the role of E2F1 has been well defined. We believe E2F2 to be of interest and have an important role in DNA repair due to its specific governance of important repair associated proteins such as STAT1. Here we present an integrative bioinformatic analysis leveraging a number of public datasets to define a key role for E2F2 in maintaining genomic integrity. Through the use of cell lines, mouse models, and human samples, we have identified that low E2F2 activity level is associated with tumors containing high levels of genomic instability. Furthermore, E2F2 status has potential clinical implications. Indeed, tumors with high E2F2 activity have an increased sensitivity to cell cycle targeted chemotherapy as well as targeted PARP inhibitors.

## Results

**Figure 1:**
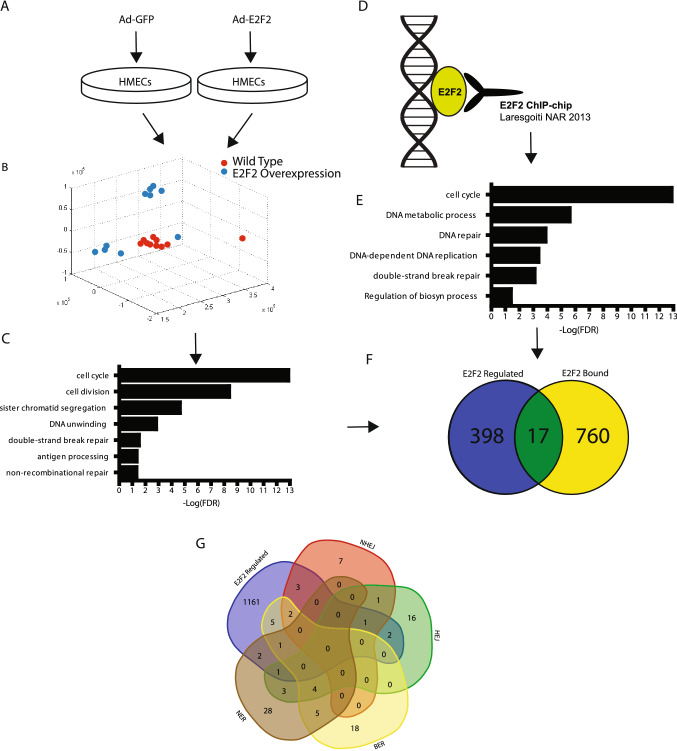
E2F2 target genes are enriched for DNA repair associated proteins. To understand genes regulated by E2F2 we utilized E2F2 overexpression data to compare the expression profile of HMECs infected with Ad-GFP and Ad-E2F2 (**A**). A graphic representation of the first three principle components reveals a consistent transcriptional response associated with E2F2 overexpression through displaying MEFs plus Ad-GFP (blue) and MEFs plus Ad-E2F2 (red) (**B**). Genes overexpressed with the addition of E2F2 as identified by SAM show a significant (FDR < 0.05) overrepresentation in key gene groups including cell cycle and repair (**C**). ChIP-CHIP of E2F2 binding genes show binding across the genome (**D**). The binding targets show a significant (FDR < 0.05) overrepresentation in a cancer related gene groups (**E**). A Venn Diagram showing genes predicted to be regulated by E2F2 from ChIP-Chip binding and over expression analysis show a small overlap in genes (**F**). E2F2 overexpressed or bound genes (E2F2 regulated) are shown to play a role in major repair pathways including Non-Homologous End Joining, Homologous End Joining, Base Excision Repair, and Nucleotide Excision Repair (**G**).

Based on the published literature for E2Fs in non-cell cycle roles, we hypothesized that E2F2 had key activities other than the traditional role in cell cycle. To test this hypothesis, we used principle components analysis on public gene expression data from mouse epithelial fibroblasts infected with adenoviral delivered GFP compared with adenoviral delivered E2F2 (Fig. 1A)^39^. This analysis revealed a consistent gene expression profile associated with over expression of E2F2 (Fig. 1B). We used Significance Analysis of Microarrays (SAM)^40^ analysis to identify significantly (q < 0.05) overexpressed genes with the infection of Ad-E2F2 relative to GFP. E2F2 induction allowed for the identification of overrepresented gene ontology groups through the use of PANTHER analysis (Fig. 1C) in the E2F2 active mouse embryonic fibroblasts. As expected, this uncovered over-representation of cell cycle proteins. Interestingly we also identified a number of repair associated gene ontology groups, including double-stranded break repair and non-recombinational repair.

To identify genes and pathways directly regulated by E2F2, we utilized publicly available E2F2 ChIP-Chip data (Fig. 1D)^41^ from T lymphocytes isolated from 4-week-old C57B16:129SV mice. This analysis utilized a mouse ref-seq promoter (− 2000 to + 500 base pairs from the transcriptional start site) to identify E2F2 bound regions. This revealed numerous gene promoters bound by E2F2 across the genome. When target genes were analyzed with PANTHER and GATHER, the ontologies were consistent with the E2F2 overexpression data. Indeed, cell cycle and DNA damage repair gene ontology groups were overrepresented, including DNA repair and double-stranded break repair (Fig. 1E) in the T lymphcytes. Surprisingly due to the different nature of the lines used in the analysis, MEFs vs T lymphocytes, we identified consistent gene ontology groups being overrepresented. However, we did not identify a large overlap in the genes identified in the overexpression of genes with the induction of E2F2 in the MEFs vs the E2F2 bound genes in the T-lymphocytes (Fig. 1F). This may indicate that the pathways are regulated by both direct and indirect/downstream targets of E2F2. To determine if there was bias towards one particular repair pathway, we identified overlap between E2F2 regulated genes (gene expression and ChIP-chip) and each of the major repair pathways including Non-Homologous End Joining, Homologous End Joining, Base Excision repair, and nucleotide excision repair (Table S1). This analysis illustrated that E2F2 regulates proteins in each repair pathway (Fig. 1G).

**Figure 2:**
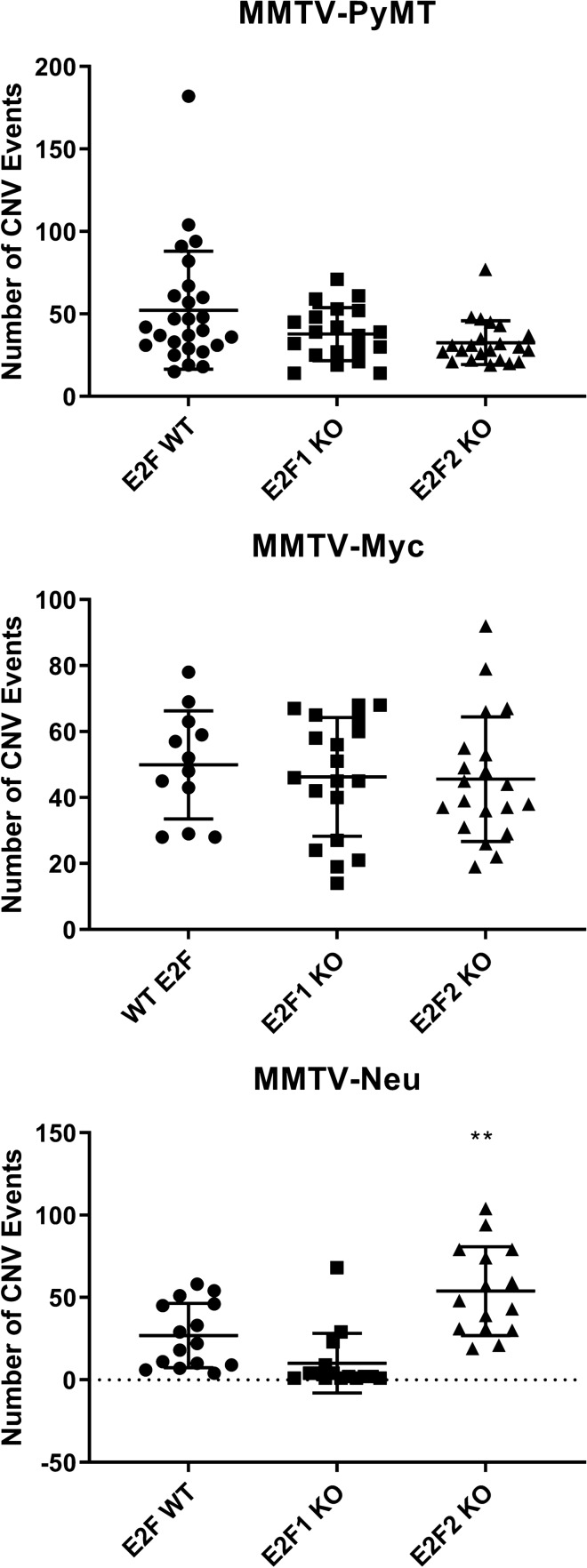
E2F2 loss is associated with higher number of copy number alterations in the MMTV-Neu mouse model. Copy number analysis obtained through the ACE algorithm for MMTV-PyMT tumor (**A**), MMTV-Myc (**B**) E2F2 knockout tumor and MMTV-Neu (**C**) models with E2F WT compared with E2F1 and E2F2 null models. This is shown to be significant across the entire genome with the E2F2 null samples having a significant increase in number of copy number event and the E2F1 null samples having a decrease in the number of copy number alterations in only the MMTV-Neu model (**P < 0.01).

To determine if there is a role for E2F2 in DNA repair processes in the in vivo setting, we utilized publicly available wildtype, E2F1, and E2F2 knockout transcriptome data within multiple genetically engineered mouse models including MMTV-PyMT, MMTV-Myc, and MMTV-Neu. This data was generated from endpoint bulk microarray analysis. We utilized ACE copy number prediction^42^ to identify copy number changes within each model. MMTV-PyMT (Fig. 2A) and MMTV-Myc (Fig. 2B) did not show a significant change in number of copy number variations with the loss of E2F1 or E2F2 compared to wildype. However, MMTV-neu showed a significant gain in the number of copy number alterations with the loss of E2F2 that was not seen with the loss of E2F1 (Fig. 2C).

**Figure 3:**
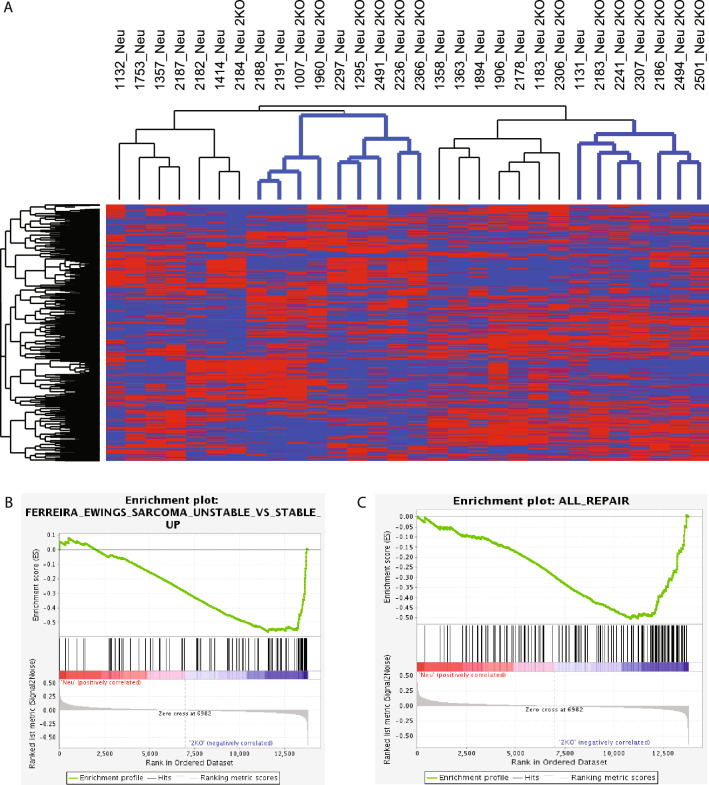
Loss of E2F2 is associated with an enrichment of genomic instability markers. E2F2 loss is associated with consistent transcriptional responses as revealed by unsupervised hierarchical clustering. MMTV-Neu or MMTV-Neu E2F2 knockout samples are arranged by column and unique genes by row. Gene expression values are represented by color from low (blue) to high (red) as indicated by the color bar. It is revealed that 2 main clusters contain an overrepresentation of MMTV-Neu, E2F2 KO samples (blue) (**A**). Consistent gene expression sets are enriched with the loss of E2F2 as identified by gene set enrichment analysis. This includes Ewings scaroma unstable vs stable up (**B**) and all repair gene sets (**C**).

To continue understanding the cause of this increased copy number variation we further investigated the transcriptomic data of the MMTV-Neu wildtype compared to the E2F2 null tumors. Unsupervised clustering identified a consistent transcriptional profile with E2F2 loss in the MMTV-Neu mouse model (Fig. 3A). This revealed five major clusters, three primarily composed of MMTV-Neu E2F2 knockout samples and two clustered primary populated with MMTV-Neu E2F wildtype samples. This demonstrated a unique gene expression profile associated with the loss of E2F2, which was unique relative to the MMTV-Neu E2F wildtype background. To explore the enriched cellular processes in this data, we used Gene Set Enrichment Analysis (GSEA) comparing MMTV-Neu tumors with and without E2F2. This revealed, similar to the in vitro data, that E2F2 null samples were enriched for both the instability gene set (Fig. 3B) and the repair gene set (Fig. 3C). We do not believe this to be in conflict with the in vitro data with an upregulated gene expression signature for repair not necessarily correlating with an increase in the repair process and may be a compensation mechanism of a deficiency of repair processes in which E2F2 target genes are upregulated by other E2F family member; however, repair is unable to occur.

**Figure 4:**
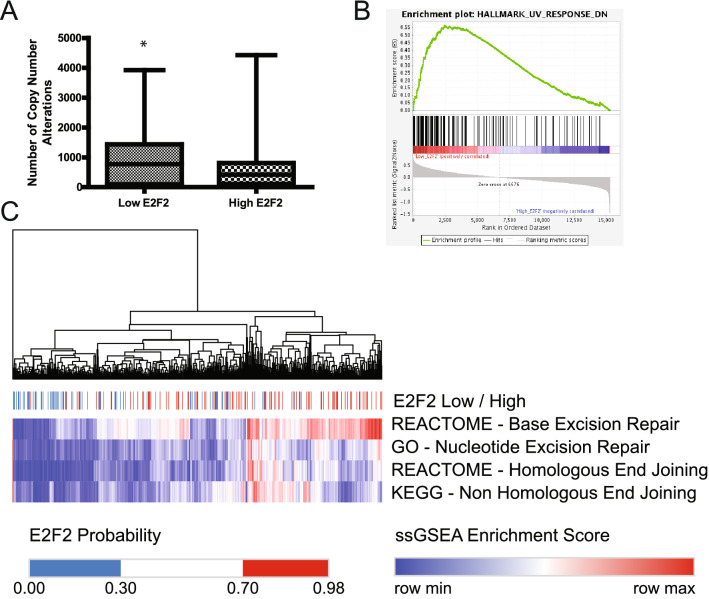
Low E2F2 activity is associated with decrease repair gene expression in the TCGA breast cancer cohort. The TCGA cohort reveals that low E2F2 activity is associated with higher number of genes with copy number alterations (**A**). GSEA analysis shows an enrichment of the Hallmark_UV_Reponse_down geneset (p < .05) (**B**). Unsupervised hierarchial clustering of ssGSEA data for TCGA breast cancer patients for each labled geneset. Scores are color coordinated with low enrichment in blue and high enrichment scores in red. Samples are labled according to low (blue) or high (red) E2F2 status (**C**).

While we have identified a potential role for E2F2 associated instability in mouse tumors, this role has not previously been examined in human breast cancer. Consistent with the mouse data we identified E2F2 high HER2 positive breast cancer to have worse overall survival (Fig. S1) as determined by log rank P-value across the KMplot dataset^43,44^. This is in contrast to all other subtypes of breast cancer which showed no correlation with overall survival. To address a potential role for E2F2 in breast cancer, an E2F2 activity signature was used to divide the TCGA breast cohort into low/high E2F2 activity groups. To create these predictions we used Ad-GFP and Ad-E2F2 infected cell lines and uses binary regression analysis to compare the TCGA data to the known adenovirus infected controls to get an activity score between 0 (low E2F2 activity) and 1 (high E2F2 activity) as previously described^39,45,46^. Consistent with the prediction from the mouse mammary tumor data, human breast cancer with low E2F2 activity contained significantly more copy number variants than those with high E2F2 activity (p < 0.05) (Fig. 4A). However, we did not identify this in any particular PAM50 subtype (Fig. S2A,B) as determined by a t-test.

Furthermore, tumors with low predicted E2F2 activity were observed to have enrichment for genomic instability in gene set enrichment analysis such as genes involved with the response to UV induced damage (Fig. 4B).

In order to determine if E2F2 preferentially regulated a specific repair pathway, we utilized single sample gene set enrichment analysis (ssGSEA)^47^. The resulting scores were low/high normalized to return an activity score between 0 and 1 for the four major repair pathways. Low E2F2 activity resulted in significantly lower activity in each repair pathway including: Base Excision Repair, Nucleotide Excision Repair, Homologous End Joining, and Non-Homologous End joining (Fig. S3). Unsupervised hierarchical clustering of this data revealed that regardless of the pathway, low E2F2 activity was associated with low ssGSEA pathway scores (Fig. 4C).

Alterations in DNA repair pathways are manifested in response to therapy. Accordingly, we sought to test whether E2F2 levels impacted therapeutic response using the CancerRx dataset. After predicting the E2F2 activity level across all breast cancer datasets we identified differentially lethal compounds between E2F2 low cell lines and E2F2 high cell lines (Table S2). This revealed a number of interesting candidate compounds. For example, tumors with low E2F2 activity responded poorly to cell cycle inhibiting compounds such as Cisplatin. However, they responded well to PIK3 targeted therapy such as PI-103. In addition, we observed that tumors with high E2F2 activity were sensitive to cell cycle compounds and resistant to other forms of therapy. This is in contrast to E2F1 in which activity was not correlated to sensitivity with any compound (Table S2). E2F1 activity is correlated to resistant to an EGFR inhibitor, HG-5-88-01, and MAPK inhibitor, ZG-10.

**Figure 5:**
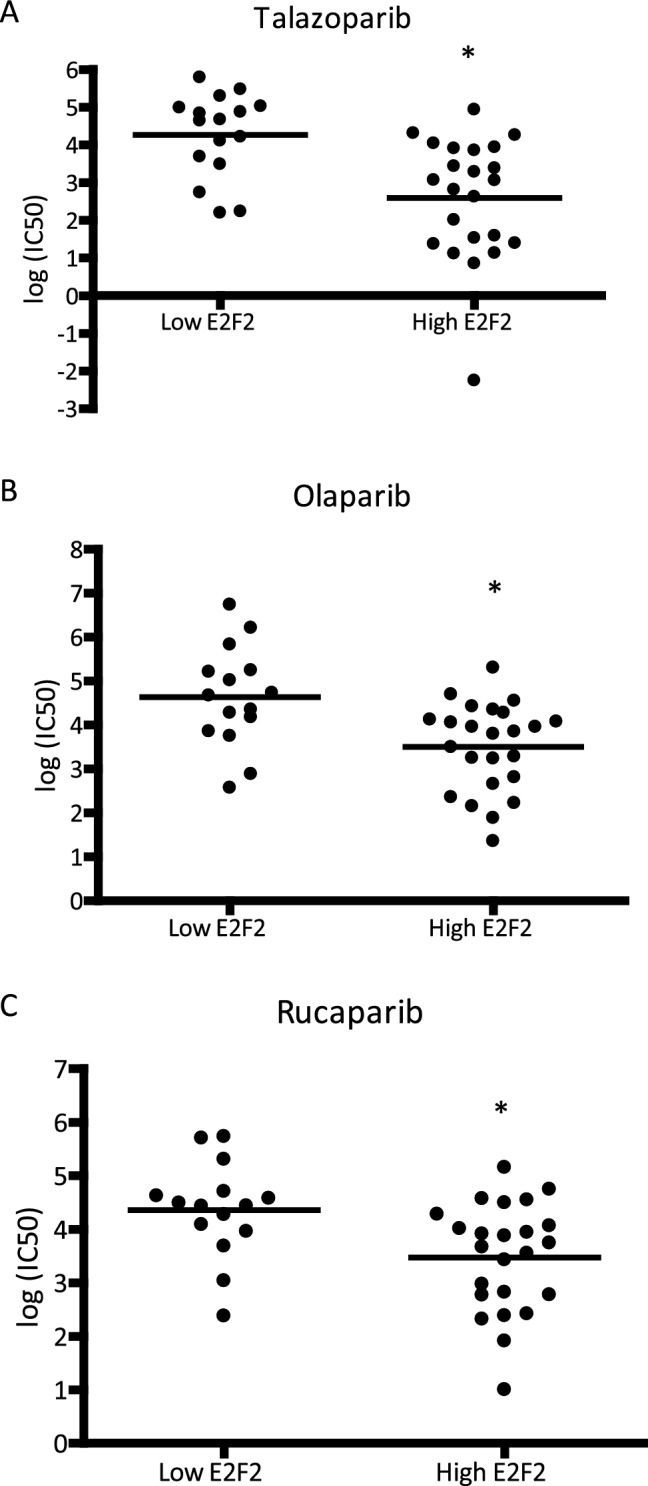
Low E2F2 activity is associated with resistance to PARP inhibitors across breast cancer cell lines. Breast cancer cell lines divided in lowly active E2F2 and highly active E2F2 show a consistent resistance to PAPRP targeted therapies associated with low E2F2 activity (p < 0.05) for Talazoparib (**A**), Olaparib (**B**), and Rucaparib (**C**).

With a role for E2F2 in repair, we examined response to repair targeted therapy, including PARP inhibitors. Surprisingly we identified that high E2F2 activity was associated with a significantly higher response to common PARP inhibitors including Talazoparib (Fig. 5A), Olaparib (Fig. 5B), and Rucaparib (Fig. 5C) as identified by a significant decrease in the IC50 of each compound on each cell line. Importantly, this correlation was independent of the BRCA1/2 status of the cell line. To test if this correlation was a function of any E2F or specific to E2F2, we performed a similar analysis for E2F1. In this analysis, we predicted E2F1 activity through the use of a gene activity signature and identified the IC50 of each cell line. Importantly, in this analysis we saw no difference in the IC50 or PARP targeted therapy in low or high E2F1 activity (Fig. S4). This indicates the identified differences in response to PARP targeted therapy are specific for E2F2.

## Discussion

In this manuscript we perform a re-analysis of publicly available data to uncover new roles of E2F2. In this manuscript we use a number of different cell types and models to identify conserved function of E2F2. Through this we identified a number of E2F2 target associated genes are involved with repair through the integration of E2F2 expression data and ChIP-Chip data. This was also investigated within the E2F2 null MMTV-null model which showed a large increase in the number of copy number alterations. Interestingly, despite their instability, these tumors showed an enrichment for an increased repair process in their transcriptome. This may be due to previously identified compensation from the E2F family members^48,49^ and merits further investigation. In total, we describe a role of E2F2 in repair and maintenance of genome integrity in both MMTV-Neu mouse model mammary tumors as well as in human breast cancer patients.

An important note is that this study was not controlled for cell cycle. Within each model it can be expected that there were a variety of cell cycle states present. It is known that E2F2 is more active in some states rather than other, so some roles of E2F2 may have been muted by the diversity of cell states. This merits further investigation.

Despite this heterogeneity, we have identified a consistent role of E2F2 in DNA stability across a combination of in silico, in vitro and in vivo models. However, due to the high-throughput nature of these datasets they have a high propensity to generate false negative or positive data. Throughout, we have utilized stringent statistical parameters to minimize these, but this should be considered when understanding this data. As with any high-throughput study, this data should be used as a hypothesis generating tool, that requires additional confirmation and mechanistic follow-up. We propose that E2F2 controls key members of many different repair pathways including HEJ, NHEJ, BER, and NER. This involvement is associated with a genomically unstable tumors with the loss of E2F2 activity in both the mouse model and the human disease.

Furthermore, this finding has potential clinical application. We have identified E2F2 activity as a biomarker for response to cell cycle inhibition therapy. E2F2 activity, as determined by a gene expression signature, correlates with PARP inhibition therapy. Interestingly, although tumors with lowly active E2F2 are significantly more unstable than E2F2 tumors they are resistant to PARP inhibitor therapy. We have identified that this effect is independent of BRCA1/2 status and is correlated with E2F2 levels. This indicates that E2F2 has a role in PARP response and loss of E2F2 may phenocopy or directly trigger other known causes of PARP inhibitor resistance. This is unintuitive and mechanistically this would be important to study. Potentially, due to the fact that E2F2 low tumors are highly unstable they have adapted to better tolerate DNA damage. As a result, they are more able to tolerate PARPi induced damage than an E2F2 high cell line.

This manuscript serves as an informatic proof of concept study for an expanded role of the E2Fs in DNA repair in breast cancer. The data presented here, combined with the previously established literature, show key roles of E2F1 and E2F2 as contributing factors in the genomic instability seen in breast cancer. Further research needs to be completed of the other E2F family members to identify if this role is unique to E2F1 and E2F2 or if other E2Fs may play similar roles. Indeed, while certain E2Fs have specific roles in mammary development^49,50^ and tumor biology^3,4,6^, there is significant overlap and compensation amongst the E2Fs^48,49^.Taken together, this data shows the key role that the E2Fs play in cancer progression and heterogeneity. As central drivers of repair and due to the impact they have on the ability of a tumor to respond to key therapies, there must be more research to understand the clinical application of E2F status and the way that it should shape patient care.

## Materials and methods

### Datasets used

For the E2F2 overexpression data, Affymetrix cDNA microarray profiled gene expression data was downloaded from previously published data^39^. This dataset consisted of adGFP and adE2F2 infected mouse embryonic fibroblasts. The data was processed using Mas5 normalization through Affymetrix expression console.

For E2F2 binding analysis we utilized ChIP-Chip for data for E2F2 T lymphocytes isolated from 4-week-old C57B16:129SV mice^41^. This manuscript identified E2F2 bound promoters through the use of a gene promoter (− 2000 to + 500 base pairs from the transcriptional start site) and processed through the use of the NimbleScan program.

For the E2F2 knockout data in the various mouse backgrounds we downloaded GSE24594^4^ GSE104397^6,51^ and GSE42533^3^ for the MMTV-Myc, MMTV-PyMT, and MMTV-Neu data accordingly. Raw data was downloaded from ncbi gene expression omnibus and processed through the use of Affymetrix expression console and RMA normalization.

### Overrepresentation analysis

All overrepresentation analysis experiments were performed through the use of GATHER^52^ and PANTHER bioinformatic analysis to identify overrepresented gene ontology groups. Significant groups were noted filtered by a p value of less than 0.05 and a Bayes factor greater than 3.

### ssGSEA and clustering analysis

ssGSEA was performed on the Broad genepattern software with the designated genesets for each repair pathway downloaded from msig-db. These scores were normalized between 0 (low) and 1 (high). Samples were clustered through the use of Morpheus (https://software.broadinstitute.org/morpheus/). Clustering was performed using Complete linkage unsupervised hierarchical clustering. Heatmaps were visualized using MATLAB.

### ACE analysis

ACE analysis was performed as previously described to infer copy number alterations from gene expression data^42,53^. The analysis was performed with default parameters and used a significance cutoff of q < 0.05.

### E2F2 activity levels

E2F2 activity was assayed using a gene expression signature as previously described^45,46,50^. Briefly this method identifies differentially expressed genes between Ad-GFP and Ad-E2F2 infected cell lines and uses binary regression analysis to compare unknown samples and known controls from each group to get a score between 0 (low E2F2 activity) and 1 (high E2F2 activity).

### Copy number analysis of TCGA cohort

For the human patient analysis, we utilized the TCGA breast cancer cohort^54^. Patients were classified as E2F2 high or low through the gene expression signature approach described above. The gene copy number changes was identified as the number of genes which had a deep deletion or high level amplification as identified by a GISTIC score of − 2 or 2. p-value was determined by t-test between low and high E2F2 activity cohorts.

### Survival analysis

For the overall survival data, we utilized the KMplot.com dataset^43,44^. Patients were identified into the E2F2 high (top 33%) or low (rest of samples) by E2F2 expression levels and classified by PAM50 status. P-value was calculated by log rank test.

### E2F2 drug sensitivity data

Breast cancer gene expression data was downloaded from the cancer cell line encyclopedia^55^. From this data E2F2 activity was predicted as described above. For drug sensitivity data, we downloaded the small molecule sensitivity dataset from CancerRx.org. Breast cancer cell lines were divided into high and low E2F2 activity groups and significantly different compounds between the two groups were identified by significantly different IC50’s as identified by a student’s T-test.

### Informed consent

This articles does not contain any studies with human participants performed by any of the authors.

## Supplementary information

Supplementary Information 1.

Supplementary Legends.

Supplementary Figure S1.

Supplementary Figure S2.

Supplementary Figure S3.

Supplementary Figure S4.

Supplementary Table S1.

Supplementary Table S2.

## Data Availability

All datasets used in this publication can be accessed on Gene Expression Omnibus through their appropriate GSE number as noted in the manuscript. This article is present on a university repository website and can be accessed on https://urldefense.com/v3/ and https://www.biorxiv.org/content/10.1101/777870v1__;!!HXCxUKc!klXu58cpO7ltAWssBJNGruwrIBog3joS5t3Ul-X4DHqhjl9G3MApailfU2FW4UQ$.
