# Supplementary material for: Low E2F2 activity is associated with high genomic instability and PARPi resistance
Source: Sci Rep. 2020 Oct 21;10:17948. doi: 10.1038/s41598-020-74877-1 (PMC7578094; doi:10.1038/s41598-020-74877-1)
Supplement: Supplementary file 1 — Supplementary Information 1. [file 41598_2020_74877_MOESM1_ESM.pdf]

Low E2F2 activity is associated with high genomic instability and PARPi resistance

Jonathan P Rennhack and Eran R. Andrechek

Supplementary Information
