# Supplementary material for: Low E2F2 activity is associated with high genomic instability and PARPi resistance
Source: Sci Rep. 2020 Oct 21;10:17948. doi: 10.1038/s41598-020-74877-1 (PMC7578094; doi:10.1038/s41598-020-74877-1)
Supplement: Supplementary file 2 — Supplementary Legends. [file 41598_2020_74877_MOESM2_ESM.docx]

**Supplemental Legends**

**Supplemental Figure 1**

Kaplan Meier overall survival plots of KMplot.com breast cancer cohort divided by Pam50 survival split by top 33% E2F2 expression (red) vs the rest of the patients (black). All patients regardless of subtype (A), Basal (B), Luminal A (C), and Luminal B (D) showed no differences in overall survival. HER2 positive patients (E) showed an decrease in overall survival associated with an increase in E2F2 expression. Y-axis of all graphs report the probability of overall survival and below the graph is the number of patients in each cohort at each time point (months). P value as determined by log rank is displayed on the figure.

**Supplemental Figure 2**

Copy number changes in TCGA cohort split by PAM50 subtype show no difference with relative E2F2 status. Absolute number (A) or mean centered (B) of copy number changes (A) of Basal, HER2 positive, Luminal A or Luminal B samples show no difference as determined by a t-test between E2F2 low and high samples.

**Supplemental Figure 3**

Dotplot representation of figure 4, split by major repair pathway. ssGSEA shows low E2F2 activity is associated with lower gene expression enrichment in a number of different repair pathways including Base Excision Repair (A), Nucleotide Excision Repair (B), and Homologous End Joining (C), Non-Homologous End Joining (D).

**Supplemental Figure 4**

Breast cancer cell lines divided in lowly active E2F1 and highly active E2F1 show no consistent resistance to PAPRP targeted therapies associated with low E2F1 activity for Olaparib (A), Rucaparib (B) or Talazoparib (C).

**Supplemental Table 1**

Table One includes a list of of E2F2 bound or co-expressed genes included in each of the major repair pathways.

**Supplemental Table 2**

Table Two includes the IC50 for various breast cancer cell lines correlation to E2F1 or E2F2 pathway signature activity. The last rows shows significance through a t-test for low (<0.5) and high (>0.5) E2F1 or E2F2 predictions. Significant compounds (p<0.05) are shown in bold.
