# Supplementary figures and images for: Low E2F2 activity is associated with high genomic instability and PARPi resistance
Source: Sci Rep. 2020 Oct 21;10:17948. doi: 10.1038/s41598-020-74877-1 (PMC7578094; doi:10.1038/s41598-020-74877-1)

A

All Subtypes

**E2F2 (207042\_at)**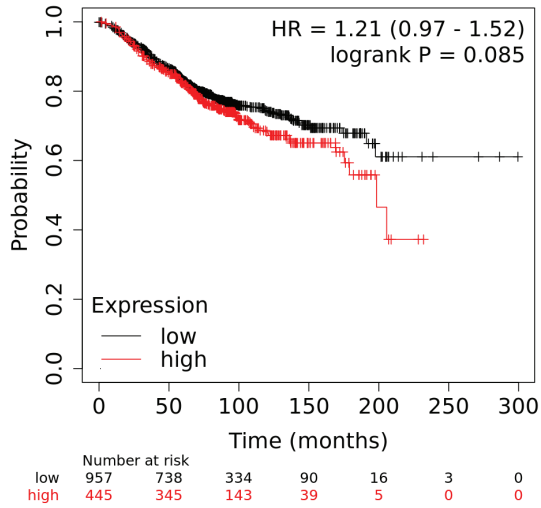

B

Basal Subtype

**E2F2 (207042\_at)**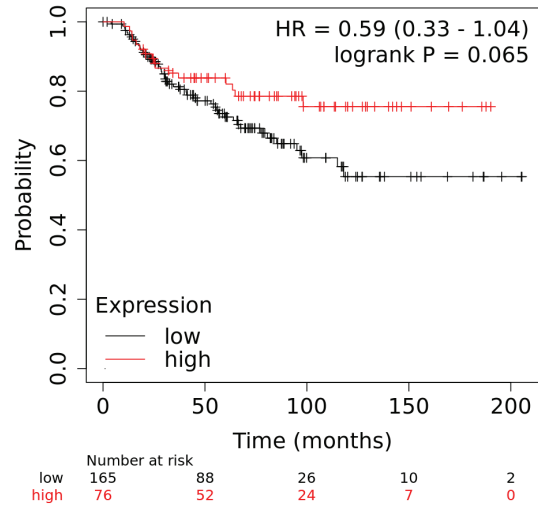

C

Luminal A

**E2F2 (207042\_at)**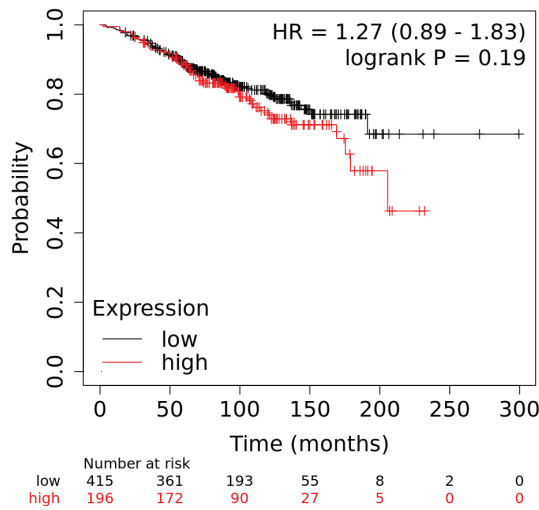

D

Luminal B

**E2F2 (207042\_at)**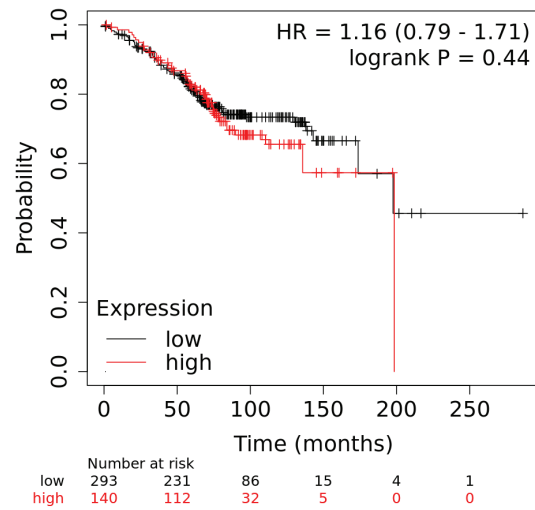

E

HER2+ve

**E2F2 (207042\_at)**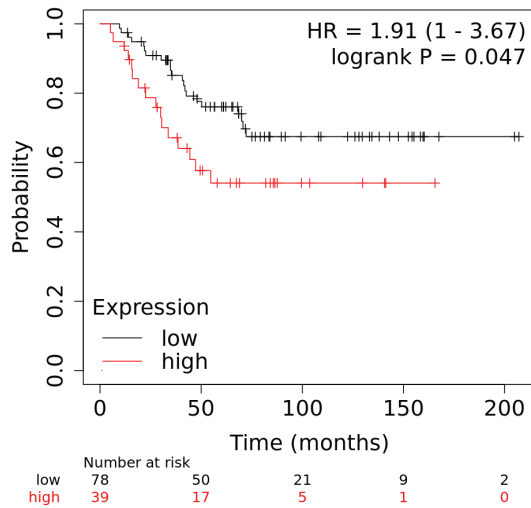

Supplement: Supplementary file 3 — Supplementary Figure S1. [file 41598_2020_74877_MOESM3_ESM.pdf]

A

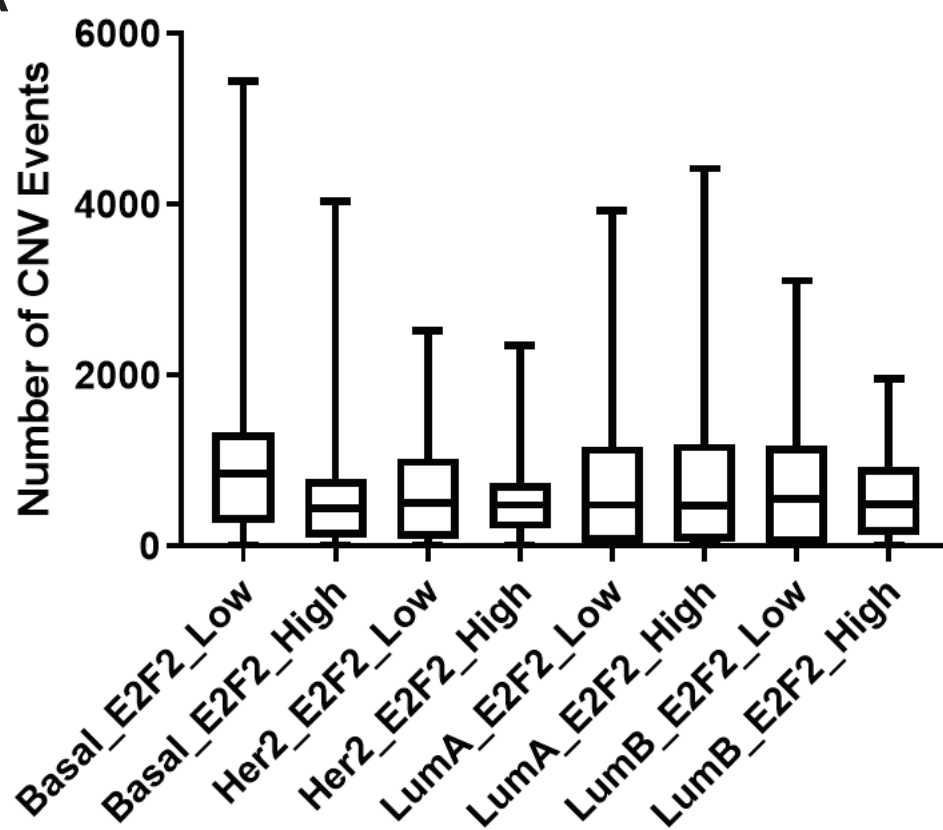

B

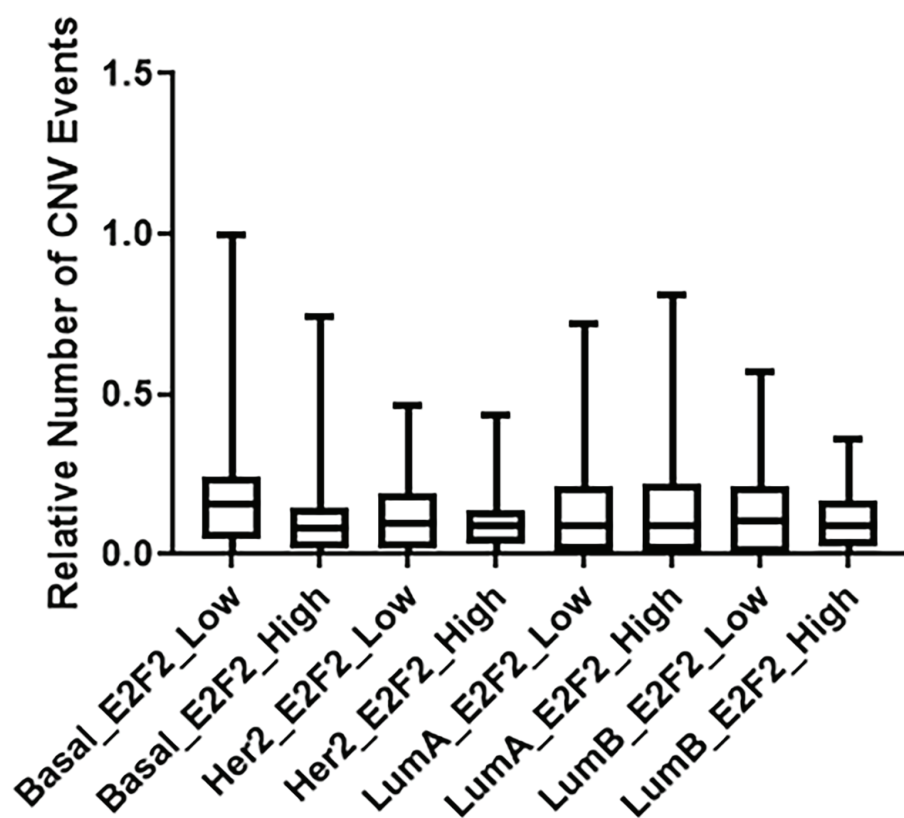

Supplement: Supplementary file 4 — Supplementary Figure S2. [file 41598_2020_74877_MOESM4_ESM.pdf]

Rennhack - Figure S3

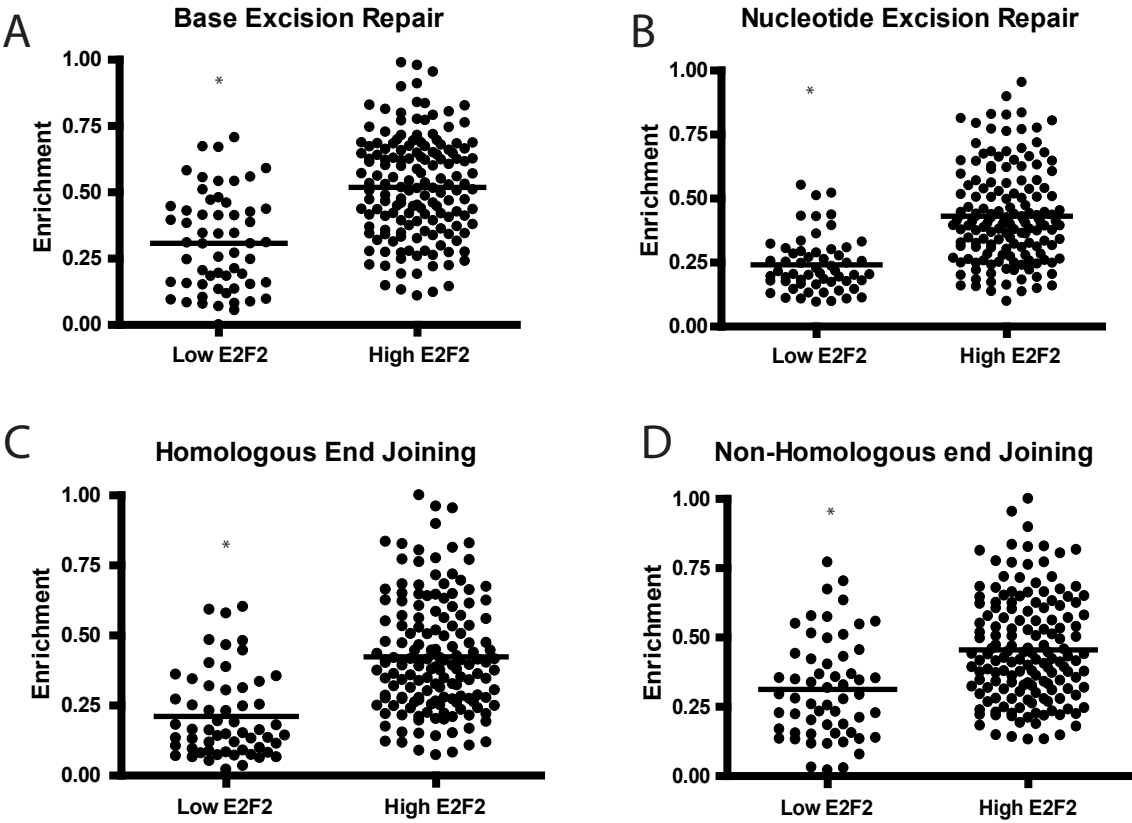

Supplement: Supplementary file 5 — Supplementary Figure S3. [file 41598_2020_74877_MOESM5_ESM.pdf]

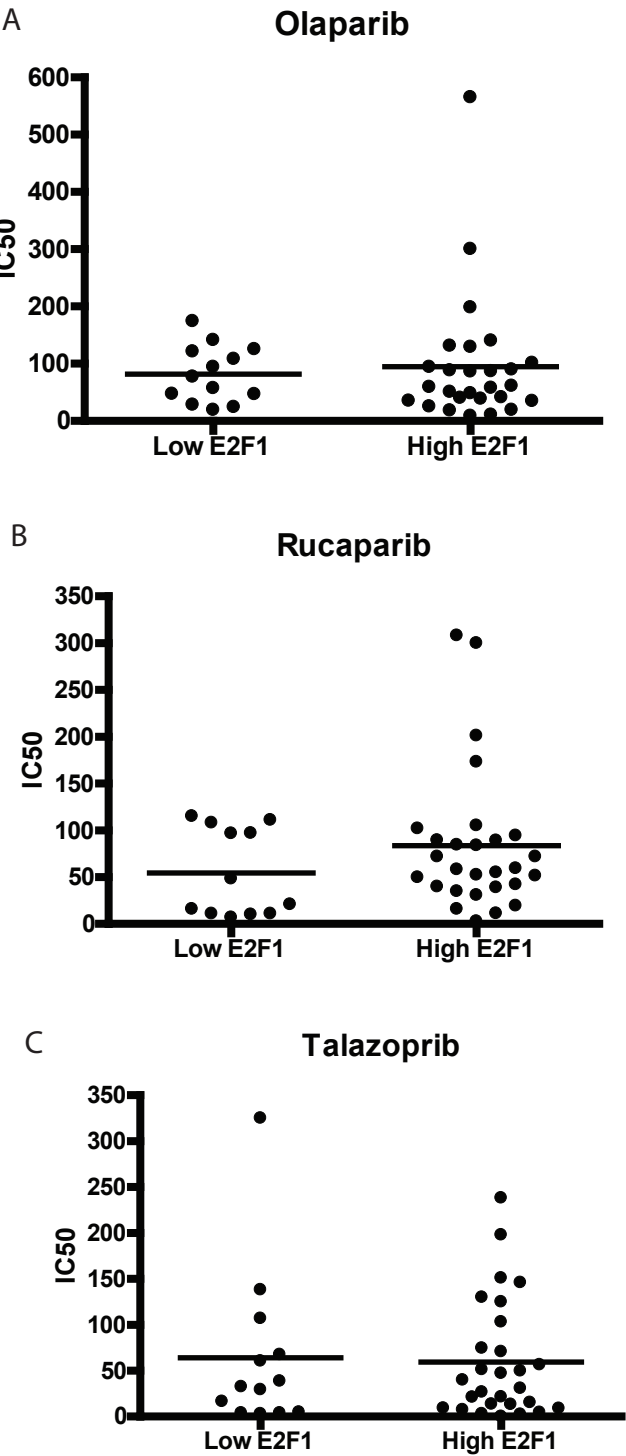

Supplement: Supplementary file 6 — Supplementary Figure S4. [file 41598_2020_74877_MOESM6_ESM.pdf]
